# Professional self-understanding of teachers in different career stages: a phenomenological analysis

**DOI:** 10.1186/s40359-022-00769-w

**Published:** 2022-03-07

**Authors:** Ersin Yağan, Mustafa Özgenel, Fatih Baydar

**Affiliations:** 1 Tuzla Public Education Centre, Ministry of National Education, Istanbul, Turkey; 2 Istanbul Sabahattin Zaim University, Istanbul, Turkey

**Keywords:** Self-understanding, Professional self-understanding, Professional development, Career stages

## Abstract

Teachers’ sense of identity plays a central role in understanding their actions as individuals inside and outside the classroom. Therefore, it is important to determine teachers’ understanding of who they are. Thus, teachers’ opinions about their professional self-understanding in the study were aimed to be analysed. Although teacher training practices are gaining importance rapidly, it is seen that studies on how teachers evaluate themselves are not sufficiently included in the literature. For this purpose, the research was carried out using the qualitative research method and phenomenology pattern to consider teachers’ opinions about their professional self-understanding holistically. The research study group consists of 44 teachers working in primary, secondary, and high schools in the Pendik district of Istanbul. Research data were obtained from in-depth interviews with teachers through semi-structured interview forms. The findings obtained were grouped under five themes: “Image, Respect, Job Motivation, Perception of Duty, and Future Views”, considering the relevant literature. The study revealed that professional self-understanding is closely affected by emotions and teachers’ social status. Considering the relationship between social status, acceptance, and individual motivation, it is thought that the negative judgments in society about the teaching profession make individuals reluctant to choose this profession. For this reason, it can be evaluated that studies should be carried out to restore the eroded reputation of the profession in society and that this profession should be provided with much more attractive opportunities economically.

**Supplementary Information:**

The online version contains supplementary material available at 10.1186/s40359-022-00769-w.

## Introduction

There is an increasing interest in studies on the importance of discovering the thoughts and feelings of teachers and their teaching practices [26–28, 39, 48, 54–56]. The main reason for this situation is that teachers play a leading role in implementing all reform or innovation initiatives, which directly impact the success of the education system, particularly student outcomes. Educational reforms and practices that do not consider teachers’ feelings and thoughts result in failure. In this sense, it is necessary to examine and know teachers’ views on their professional self-understanding.

An important prerequisite for responding to the needs of others and connecting with them is a sense of self-understanding [7]. Self-understanding is an individual’s recognition of their strengths and weaknesses, needs, impulses, feelings and thoughts, and the understanding one has about “self” [40]. Professional self-understanding is the awareness of how past experiences and current situations affect them and how one sees oneself as a professional [47]. The concept of professional self-understanding is important for the professional life of teachers because how teachers experience, make sense of, interpret, and position themselves in their profession helps them understand and recognise themselves, shaping and developing their professional lives. Additionally, understanding teachers’ professional self-understanding supports professional learning and increases the quality of educational practices [6]. In other words, defining and grasping teachers’ professional self-understanding can serve as a powerful tool to improve our understanding of how teachers behave in their professional lives, recognising their strengths and weaknesses, and the gap between their actions and thoughts.

Professional self-understanding is shaped throughout the professional life process [6]. The professional self-understanding of teachers is shaped in the career process. In this sense, the professional self-understanding of teachers consists of two parts: pre-service and in-service. Pre-service generally refers to the undergraduate program offered by the university. On the other hand, in-service refers to the time elapsed between a teacher’s first entry into the profession and leaving the profession for different reasons. During this period, teachers’ attitudes towards the profession, perceptions, motivations, expectations, perspectives, and professional knowledge and skills change by being affected by various factors. Furthermore, teachers’ positive feelings and thoughts about their profession, being understanding towards the problems they encounter in their professional life, and accepting the negativities as a part of their professional life (in other words, having a strong professional self-understanding) can strengthen them because professional self-understanding increases the probability of one’s actions for reaching the intended result [6]. Whether the concept of self-understanding, which is seen to be very important for teachers, manifests itself in a certain standard or a variable form during the professional life of teachers is a question that needs to be answered. When a teacher continues their professional life for a long time in the developing and constantly changing information age, it can be thought that they exhibit very different attitudes and behaviours from their career starting point. For this reason, examining the professional self-understanding portraits of teachers in different periods of this professional career journey that they draw for themselves can support the professional development of teachers, increase their performance, contribute to the development of the teaching profession, and improve our understanding of the quality of education. This article presents and discusses a perspective on the professional self-understanding of in-service teachers at different career cycles/career stages.

### Professional self-understanding

The concept of professional self-understanding was defined by Kelchtermans [36]. Professional self-understanding is conceptualised by analysing teachers’ knowledge and experiences [7]. According to Kelchtermans [40], self-understanding is achieved by clarifying the individual’s understanding about themselves or others. Professional self-understanding is teachers’ self-interpretation and understanding of themselves as a teacher [36–39]. Kelchtermans analysed educators’ career stories and identified five components that make up professional self-understanding. These components are; the self-image of the teacher (descriptive), the teacher’s self-esteem (evaluative), the job motivation of the teacher (behavioural), the task perceptions of the teacher (normative), and the teacher’s perspective on the future.

- *Descriptive component* The teacher’s self-image is descriptive and represents how teachers define themselves. Self-image is based on self-perception but is largely related to what others reflect on teachers. A teacher’s self-image is strongly influenced by how other people perceive them.
- *Evaluative component* The teacher’s self-esteem is closely related to self-image and is an evaluative component of self-compassion or self-esteem. It refers to teachers’ evaluation and appreciation of their actual job performance. Feedback from others is important in self-esteem. However, they are filtered and interpreted. Some are considered more valuable or important. This component, which evaluates the teacher’s performance, expresses their appreciation of their actual job performance.
- *Behavioural component* The job motivation of the teacher refers to the motives and impulses that cause people to choose to become, remain, and leave the profession. In addition to the teacher’s working conditions, the perception of duty that enables them to act according to a normative program also determines work motivation.
- *Future perspective* Self-understanding also encompasses the future perspective that reveals one’s expectations for the future. The teacher’s perspective on the future also includes their expectations from their professional life. This perspective emphasises the dynamic nature of self-understanding. Individuals’ present actions are influenced by their meaningful past experiences and future expectations.
- *Normative component* The teacher’s task perceptions cover their views on what constitutes the professional work program and their duties and responsibilities to do their job well. The perception of duty constitutes the normative basis of teacher judgments and decisions, which have moral consequences as it affects the lives and needs of students for whom the teacher is responsible and feels responsible [25, 36–41].

These five components of professional self-understanding can be distinguished analytically. However, they are all intertwined and refer to each other. In this sense, professional self-understanding is both an inclusive (integrative) and analytical (differentiated) concept. Thus, it provides an insight into the dynamic nature and contextual entanglement of teachers’ sense of self. This perspective provides a conceptual, analytical tool for revealing the factors that affect all aspects of teaching the “self” [40, 41].

### Teachers’ career stages

Researchers, who have been examining teachers’ professional development processes in the pre-service and in-service periods since the early 1970s, realised that teachers showed different development and suggested different models for career stages. The main purpose of determining the professional development stages of teachers is to identify the problems encountered in professional development, to guide them to overcome these difficulties, and ultimately to increase the performance of teachers in the classroom and the quality of education [14].

The career concept is defined as “the individual’s step-by-step and continuous progress in any business area throughout his working life, gaining experience and skills” [2]. Burden [11] determined that teachers differ according to their career stages. Steffy and Wolfe [51] state that as teachers progress throughout their careers, they think critically about their professional practices, redefine their assumptions and beliefs, and engage in transformational processes by making self-evaluations. Since teachers have different job/professional knowledge, skills, competencies, behaviours, and attitudes and experience different emotions, they experience a cycle throughout their careers, influenced by personal, organisational, and environmental factors at different periods [11]. However, as teachers develop professionally, specialise in their fields or gain new perspectives, they transition between various career stages [2]. Teachers may spend more or less time in each stage, even if they have similar developmental-mental characteristics as other colleagues [11]. In other words, teachers’ career cycles do not “automatically” move forward [58].

Teachers differ in their professional/job skills, knowledge, behaviours, attitudes, perspectives, and professional activities at different points in their careers [11]. For this reason, different models ranging from 3 to 8 stages have been proposed to explain teachers’ career stages. While creating these models, teachers’ professional interests and professional development needs were considered, and it was determined that each teacher had different needs at some stages and the time/duration they spent differed [4, 11, 12, 14, 17, 19, 22, 23, 29, 34, 57]. These models differ in terms of the number of stages, how these stages are defined, and whether teachers act linearly between stages [21]. According to Huberman [29] teachers’ career development is shaped by influencing personal experiences, social environment, and organisational factors. For this reason, Fessler and Christensen [22] argue that teachers’ career cycles are not linear; they reflect their reactions to these factors, progress through different stages, and can even skip certain stages. In other words, a teacher can switch from one stage to another or return to the previous stage [21]. Even a teacher at the end of their career may revert to being a novice teacher if faced with an entirely new teaching assignment, or a teacher may not automatically become a professional teacher after serving five years as an apprentice teacher [58].

It is necessary to state that examining teachers’ professional self-understanding, which reflects their feelings and thoughts, has an important and central role in understanding their professional actions inside and outside the classroom to adapt to the changes experienced today. Therefore, teachers’ self-understanding about who and what they are is considered important [40]. Determining and understanding their professional self-understanding can contribute to the development of the teaching profession and increase the quality of education. Therefore, teachers can cope with the personal problems they face and respond to the expectations of change and innovation of society the political environment. It is also important to make a process-oriented analysis of whether teachers’ self-understanding differs in career stages. From this point of view, the research aims to analyse teachers’ opinions at different career stages on their professional self-understanding.

## Methods

### Research design

The qualitative research method was used to holistically address teachers’ views on their professional self-understanding. The research was carried out according to the phenomenology design. Phenomenology is a qualitative research design in which the researcher tries to understand and explain how one or more participants experience a phenomenon. Here the answer to the following question is sought: “What is the meaning, structure, and essence of a phenomenon for the individual?” [15]. Phenomenology is used for studies that aim to investigate the phenomena that we frequently encounter daily, that are not foreign to us but that we cannot fully comprehend, and constitute a suitable research ground [66]. In this study, the phenomenology pattern was adopted to reveal how teachers make sense of themselves professionally and how they position themselves.

### Participants

The participants consist of 44 teachers working in primary, secondary, and high schools located in the Pendik district of Istanbul. In determining the study group, the maximum diversity sampling type was employed. By choosing this sample type, it was aimed to capture a deep understanding of professional self-understanding by reflecting the diversity of teachers with different seniority degrees to the maximum sample. According to Patton [50], when selecting a small sample with a large variety, data collection and analysis will reveal two findings: (1) high quality and detailed explanations useful to clarify the uncertainties and diversity of each case and (2) presents common patterns at which important issues intersect and reveal their importance.

Bakioğlu’s (1996) study determined 5 stages following the career stages suggested for teachers to examine their thoughts on the concept of professional self-understanding (since all participants were actively working, the 6th phase, known as the ‘retirement phase’ has been removed). Accordingly, between 1 and 5 years (N = 6) professional seniority was "entering the career" phase, 6–10 years (N = 7) "settling" phase, 11–15 years (N = 6) "experimentalism" phase, 16–20 years (N = 11) "expertise" phase, and 21 years and above (N = 14) professional seniority was the "calm" phase. 20 of the teachers are males, and 24 of them are females. While 32 of the teachers are undergraduates, 12 are master’s graduates. Information about the participants is given in Table 1.

**Table 1:** Demographic characteristics of the participants

Participants	Gender	Educational status	Age	Seniority
P1	Male	Master’s Degree	51+	21+
P2	Female	Undergraduate	31–40	16–20
P 3	Male	Undergraduate	51+	21+
P 4	Female	Master’s Degree	41–50	21+
P 5	Male	Undergraduate	51+	21+
P 6	Male	Undergraduate	41–50	21+
P 7	Male	Undergraduate	41–50	16–20
P 8	Female	Undergraduate	41–50	21+
P 9	Female	Undergraduate	31–40	11–15
P 10	Female	Undergraduate	31–40	11–15
P 11	Female	Undergraduate	− 30	− 5
P 12	Male	Undergraduate	41–50	21+
P 13	Female	Undergraduate	41–50	21+
P 14	Male	Master’s Degree	41–50	16–20
P 15	Male	Master’s Degree	41–50	16–20
P 16	Male	Master’s Degree	31–40	6–10
P 17	Female	Undergraduate	51+	21+
P 18	Female	Undergraduate	41–50	16–20
P 19	Female	Undergraduate	− 30	6–10
P 20	Female	Undergraduate	31–40	− 5
P 21	Female	Undergraduate	31–40	6–10
P 22	Female	Undergraduate	41–50	16–20
P 23	Female	Undergraduate	41–50	16–20
P 24	Male	Master’s Degree	31–40	6–10
P 25	Male	Undergraduate	31–40	6–10
P 26	Male	Undergraduate	− 30	− 5
P 27	Male	Undergraduate	41–50	16–20
P 28	Female	Master’s Degree	41–50	16–20
P 29	Female	Undergraduate	31–40	11–15
P 30	Female	Master’s Degree	− 30	− 5
P 31	Male	Undergraduate	41–50	21+
P 32	Female	Master’s Degree	31–40	11–15
P 33	Female	Undergraduate	41–50	− 5
P 34	Male	Undergraduate	31–40	6–10
P 35	Male	Master’s Degree	31–40	− 5
P 36	Female	Undergraduate	41–50	21+
P 37	Female	Undergraduate	41–50	21+
P 38	Male	Undergraduate	31–40	11–15
P 39	Female	Undergraduate	31–40	6–10
P 40	Female	Master’s Degree	41–50	21+
P 41	Male	Master’s Degree	31–40	16–20
P 42	Male	Undergraduate	31–40	11–15
P 43	Female	Undergraduate	51 +	21+
P 44	Male	Undergraduate	41–50	16–20

All participants were given in-depth interview codes indicating some of their characteristics, which were used in the study. For example, the participants were coded as follows: Order No—Gender—Age—Educational Status—Seniority = 8F43MD21 (8th Rank—Female teacher—43 years old—Master’s Degree—teacher for 21 years).

### Data collection tool

In-depth interviews were used as a data collection tool to reveal the teachers’ views on their professional self-understanding. The interviewee is asked a series of questions. The purpose of using a semi-structured interview form is to enable the participants to answer questions with fixed options and examine the phenomenon in depth. The interviews were conducted at the schools where the teachers work, and each interview lasted an average of 25 min. Since the interviews were conducted with Turkish teachers, the interviews were conducted in Turkish and audio-recorded with their permission. In line with the main purpose of the research, answers to the following questions were sought.

1. How do teachers in different career stages describe themselves as teachers (professional image)?
2. How successful do teachers at different career stages find themselves professionally (self-respect)?
3. What are the reasons for teachers in different career stages for choosing the profession (job motivation)?
4. What do teachers at different career stages do to be successful/good professionally (sense of duty)?
5. What are the future professional career goals of teachers in different career stages (future vision)?

### Data analysis

Descriptive analysis was performed to reveal the themes from the qualitative data obtained from the interview. Content analysis was performed to determine the sub-themes and codes. Analyses were carried out with the joint contribution of three researchers. Content analysis aims to reach concepts and relationships that explain the collected data. After defining the concepts related to the phenomenon and determining the units of analysis, data reduction was performed. Sub-themes and codes (descriptive and interpretative) were determined to examine and evaluate the data more closely [20].

## Findings

There are 5 themes obtained as a result of the analysis. These are “Image, Respect, Job Motivation, Task Perception, and Future Vision”.

**Fig. 1:**
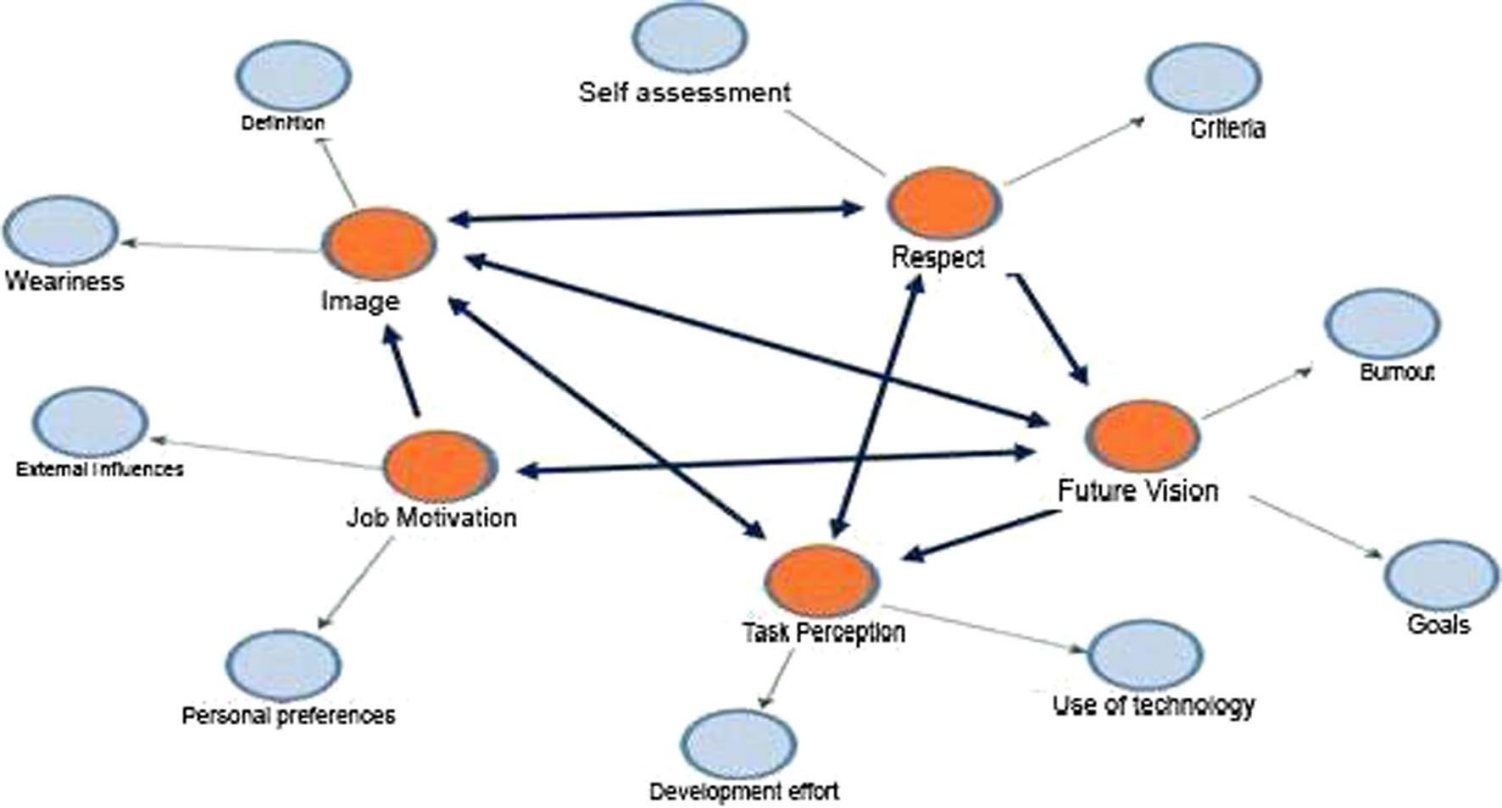
Relationship between themes of teachers regarding professional self-understanding

As seen in Fig. 1 above, when the findings are examined, the participants’ opinions about how they evaluate themselves as teachers affect their professional practices and future planning.

### Professional image

Data collected from the participants were evaluated, and findings regarding the Image Theme were analysed under the subthemes of “Definition and Weariness”.

#### Definition

Participants were asked what kind of teacher they consider themselves to be. Participants in response to this question stated to find themselves as; Innovative, open to development (f = 14), selfless, devoted, loves the profession (f = 7), disciplined (f = 7), researcher, productive, hardworking (f = 6), open to communication (f = 6), kind-firm (f = 4), equitable (f = 3), has self-improvement goals (f = 3), idealistic (f = 2), empathetic (f = 2), and achievement-oriented (f = 1).

“I define myself as a teacher who **researches, is open to innovations**, learns while teaching, enjoys his work, is open to communication, loves to produce, and aims to bring his student to a level that is self-sufficient in any environment” (13F45UD21).

“I am a **selfless and devoted** teacher. I do not come to the lesson unprepared. When I have a student who does not understand a topic, I do not hesitate to teach them repeatedly. I worry about the troubles of each of my students. I financially and morally support my students who are in need” (5M52UD23).

“I am proficient, have good risk and classroom management, **disciplined**, know and apply the regulations, stand at an equal distance to students, look objectively to students, do my job properly and on time, attend class on time, making sure students do not feel inadequate, and I am sincere towards students” (36F48UD23).

“I like to **research and produce**. I also force my students to do research. I **like working**, not intelligence” (1M56MD25).

“I am a teacher who is **open to communication**, acts in a planned way, has goals**, pays attention to communication** with parents and students, tries to get students gain good behaviours as well as transferring academic knowledge” (31M59UD28).

“I am a teacher who tries to **behave equally** to her students and constantly strives to complete the goals I set” (15M44MD18).

“…I am a teacher trying to **improve myself**” (18F43UD19).

“I am an **idealist** teacher. I want to contribute to the success of the country, family, and children” (3M51UD30).

“I am a **kind-firm** teacher. I am compassionate and loving, but when it comes down to it, I also make rules” (8F49UD27).

“**Achievement-oriented** teacher that wants to see teachers who love their profession and see more respectful students and parents” (24M32MD10).

#### Weariness

Interestingly, teachers express themselves in different ways. However, what was more interesting was the participants’ negative expressions in addition to their positive expressions. There have been teachers who stated that their professional excitement decreased, they were hopeless, they saw that the value given to them by society decreased and that this situation affected them negatively.

“I’ve been a teacher for 20 years. My **excitement has decreased**” (22F42UD19).

“An idealistic teacher who struggles not to become hopeless as the value given to teachers decreases day by day” (32F34MD12).

“I try to do my best but sometimes feel like I am failing and **have feelings of futility** ...” (18F43UD19).

### Self-respect

Data collected from the participants were evaluated, and findings regarding the Respect Theme were analysed under the subthemes of “Self-assessment and Criteria”.

#### Self-assessment

When asked how successful they found themselves as a teacher, the participants responded with the following; successful (f = 28), unsuccessful (f = 7), and mediocre (f = 3). It is seen that the majority of participating teachers consider themselves to be successful. The rate of teachers who see themselves as successful corresponds to 64% of the participants, and the rate of teachers who see themselves as unsuccessful corresponds to 16%. One out of every five participating teachers did not comment on this issue.

“I find myself quite successful in my profession. I think I am competent in my field because of my experience. For this reason, I work as a visiting lecturer at the university once a week, and I am also included in various projects by authorities” (40F49MD28).

“I **don’t find myself successful** at all” (3M51UD30).

“… I am a **mediocre** teacher” (1M56MD25).

In addition, 4 participants stated that there are no concrete indicators of success in educational institutions. They stated that they see this concept as relative and did not evaluate whether they are successful.

“I cannot evaluate myself about success. What I see as **success might be different for someone else**” (4F46MD25).

“Success is a **variable concept**. It depends on what we have achieved” (38M34UD13).

#### Criteria

Feedback from students and parents (f = 7), ability to academically teach what is required (f = 6), influence students (f = 4), raise individuals who are beneficial to their country, humanity (f = 3), and fulfil the requirements of the profession (f = 2) were the criterion in evaluating the success of participating teachers. One teacher also claimed that the criterion for success was the teacher’s personal development. Interestingly, only half of the participating teachers listed criteria for success, whereas the other teachers expressed their success without putting forward any criteria.

“… **The positive feedback from parents and students** makes me say I am good at my profession now” (13F45UD21).

“I am **successful in knowledge transfer and exams**. TYT, AYT etc. develops students in the fields” (27M44UD20).

“… I **try to influence my students**, I try different methods to make them love my lesson, and direct them to absorb the content of the lesson” (2F38UD17).

“To **train people who are beneficial to the country, the nation, and humanity**. I think that counts as success” (7M41UD19).

“I find myself successful as I **complete the requirements** of my profession adequately” (12M49UD28).

“I evaluate my success in the profession not through my colleagues or student success, but by **improving myself and what I add to my knowledge** every year” (29F32UD11).

### Job motivation

Data collected from the participants were evaluated, and the Job Motivation Theme findings were analysed under the subthemes of “Personal Preferences and External Influences”.

#### Personal preferences

The teachers were asked, “Why did you choose the teaching profession?”. To the question, they stated that they act in line with their wishes and dreams by giving the answers: being beneficial to students by influencing them (f = 15), my love for children (f = 7), my childhood dream, and my admiration for a teacher (f = 2).

“… When I started my teaching profession, I felt a part of education and thought I would be a successful teacher in the future. **Influencing students, teaching them something new**, and their feedback is what motivates me the most ” (30F25MD4).

“I chose this profession because **I love children**. I love teaching people” (9F33UD12).

“It was **my childhood dream**. There are 4 teachers in my family” (19F28UD7).

“I decided to be a teacher in primary school. **I admired my classroom teacher and imitated him**” (6M49UD28).

#### External influences

Teachers responded to this question as mandatory/compulsory reasons (f = 8), the guidance of family elders (f = 6), and job guarantee-economic reasons (f = 4). It is noteworthy that almost half of the participating teachers (f = 18) stated that they did not choose the teaching profession as their primary goal in line with their wishes. They mostly evaluated it financially, considering that the main reason underlying their family’s guidance into this profession is based on a job guarantee.

“… It was **necessary** as I was assigned from a different field” (15M44MD18).

“In our time, there were not many options with a **job guarantee**” (22F42MD19).

“I **chose it because of what my mother said**. She said, “If you do not practice this profession, you will not be able to find teachers for your children either. Your profession is very important for the country” (17F53UD32).

“It was not a planned choice. Let’s say it was **fate**” (7M41UD19).

### Task perception

Data collected from the participants were evaluated, and findings regarding the Task Perception Theme were analysed under the subthemes of “Development Effort and Use of Technology”.

#### Development effort

Participants answered the question about what they do to be a successful/good teacher as follows: do research and follow good examples (f = 25), attend training (f = 10), and do self-evaluation (f = 3).

“I **research** lecture techniques. I am in search of trying to be a better narrator” (5M52UD23).

“I **follow good examples** on social media. I benefit from the ideas and practices of my experienced colleagues” (6M49UD28).

“I am a teacher open to change and developments. I always keep my communication channels open. I am trying to **benefit from professional development seminars**. I am doing a **master’s degree** in my field to update and increase my knowledge” (7M41UD19).

“**I face my shortcomings**, I accept change, I try to teach more, not know more” (14M48MD16).

#### Use of technology

Some of the participating teachers (f = 8) stated that technology is now a requirement of their profession, and therefore they do not neglect it. Most teachers state that they share information and good examples related to their fields with their colleagues, especially through social media, showing that social media is a new and powerful medium in teacher education.

“I **follow good examples on social media**. I benefit from the ideas and practices of my experienced colleagues” (6M49UD28).

“Although I do not like technology, I **try to follow all technological developments** to establish a close bond with students” (41M37MD16).

### Future vision

Data collected from the participants were evaluated, and findings regarding the Future Vision Theme were analysed under the “Goals and Burnout” subthemes.

#### Goals

When asked questions about their career goals, teachers responded by saying, “continuing as a teacher (f = 16), pursuing an academic career (f = 9), working as an administrator/manager (f = 5), writing (f = 3), and going abroad (f = 1)”. Remarkably, less than half of the participating teachers stated that they set a career goal for themselves, and the number of teachers who would not be considered old in this group was high.

“I am a teacher. I will **always remain a teacher**” (28F41MD17).

“I **want to do a master’s degree**. I am now participating in continuous pieces of training related to my profession” (11K24L3).

“I think I can change the learning environment as a **school principal**” (20F31UD5).

“I want to **write stories** for children in the future. This way, I will continue to be beneficial to my country and nation” (8F49UD27).

“My future career goal is to **live abroad for a while to learn teaching practices** and to apply them when I return to the country” (34M33UD10).

#### Burnout

Interestingly, 8 teachers stated they had no professional goals; 2 of these teachers are over 50 years old, and the other 6 participants are too young.

“**I have no career expectations**. I would be glad if I could be useful to those around me during this time” (1M56MD25).

“**I have no career expectations**” (10F34UD12).

## Discussion and conclusion

Today, many new educational practices are emerging, and it is necessary to adapt to changing practices. It can be seen as a prerequisite for teachers to discover their professional self-understanding, know themselves, adapt to changes and developments, and develop and succeed in professional life. In this sense, examining the concept of professional self-understanding can give us insight into improving the teaching profession. Kelchtermans [36, 37] argues that teachers’ emotions should be understood to understand the teaching profession based on his narrative-biographical study with teachers. It emphasises that teachers play a vital role in “understanding themselves” in coping with the challenges posed by educational reforms. For this reason, in our research, we tried to reveal how teachers make sense, position, evaluate themselves professionally, and what kind of behaviours they adopt to increase their qualifications.

It has been observed that teachers define themselves mostly as “innovative and open to development” in self-image. In addition to the developments in their fields, the teachers stated that they made an effort to learn different applications that they could use in educational activities, especially in the technological field. Teachers state that they try to adapt to innovation and prefer to work with school administrators who are open to innovation [24, 32, 44, 45, 60] and that they benefit from new teaching methods and techniques [3, 16, 35]. Self-image is the way teachers describe themselves as teachers. This image is based on self-perception and comments from students, parents, colleagues, and principals. Therefore, self-image is strongly influenced by how others perceive a person [41]. As a part of their profession, teachers are aware that they are open to innovations, their role as a pioneer in society, and the use of technology, which is a requirement of today’s information society. For this reason, it is important to see that teachers consider being open to innovations as an important part of their profession, regardless of how much this perception is reflected in practice. Teachers who received postgraduate education and were in their careers’ entry, calmness, and experimentation phases expressed themselves as open to development and technology. Remarkably, teachers in the expertise and calmness phase differ from teachers in other phases. This situation can be interpreted as teachers whose professional seniority is too advanced begin to see themselves as professionally competent and close themselves to learning new things. However, considering the dynamic nature of education, it can be evaluated that these attitudes in the expertise and calm phase harm themselves and the institutions they work for. The positive view of postgraduate teachers on this issue also reveals that the academic education of teachers should be supported. Again, among the participating teachers, it was observed that especially the teachers in the career entry and calmness phases defined themselves as disciplined. Based on these findings, it can be said that teachers who have just started their profession keep their efforts to establish dominance in the classroom at the forefront of their professional life.

Considering that the teaching profession is often associated with self-sacrifice, it is important to reveal teachers emotions when choosing their profession and the factors that affect their decision-making mechanisms. In the research, it has been seen that half of the teachers can make individual decisions in terms of self-esteem in the process of choosing the profession, and the most effective emotion is “the desire to be useful to children and humanity”. For most teachers, students are the first and most important source of feedback; teachers exist because of students. This understanding contributes to teachers’ educational practices [41]. It has been reported that teachers with high self-image and self-esteem are very motivated to implement changes/innovations themselves, are willing to implement the proposed developments/innovations, and help students and give them more responsibility for the learning process [1]. It is noteworthy that teachers in the career entry and calmness phases express this situation. It can be thought that teachers who have just started their profession start their careers with the enthusiasm of helping children. Teachers in the last period of their profession expressed the same feeling by considering the students they had trained over the years. In other words, while the teachers in the career entry phase express this feeling with the dreams of their future students, the teachers in the calmness phase express this feeling by evaluating the students they have trained over the years. In addition, it was determined that half of the participating teachers were under the influence of environmental factors when deciding on their choice of profession. They mentioned financial opportunities as the most important criterion they considered in this process. Along with studies stating that the teaching profession loses its external attractiveness due to difficulties in finding a job, such as the problem of teachers who cannot be appointed over time [13], many studies are stating that economic and external factors are among the most determining factors in teachers’ career choices [5, 10, 43, 53]. This is in line with our research findings. The common opinion of teachers in all career stages is that economic reasons and job guarantee are among the most important criteria for choosing the profession. In this context, it is seen that the holiness of teaching, which is frequently mentioned in social platforms in Turkey, is still the most dominant criterion in choosing the profession. However, the role of teachers’ economic capital, which directly affects their cultural and symbolic capital, in choosing a profession should be considered. The decline in social status, which is a manifestation of these forms of capital, may cause a decrease in interest in the profession.

Although the criteria and measurement of success in education is a very difficult subject, how successful teachers see themselves professionally is very important. After making this evaluation, it is expected that a teacher will try to improve themself and ensure the continuity of their professional development. It was observed that the majority of the participating teachers evaluated themselves as professionally successful in terms of job motivation. Teachers evaluate success mostly through the verbal feedback they receive from students and their parents. Work motivation refers to the motives or impulses that push people to become, continue, or quit teaching [41]. Many studies show that teachers consider themselves professionally competent and have professional self-confidence [8, 9, 18, 33, 46]. It has been determined that teachers who are open to innovation have increased work motivation due to the successful implementation of innovations. Whereas teachers who have difficulty accepting innovation experience inadequacy in transferring sufficient knowledge to students [1]. It is noteworthy that the few teachers who consider themselves unsuccessful among the participating teachers are in the expertise and calmness phase. This situation can be interpreted as teachers putting themselves into a clearer professional accounting after working for a long time, unlike young teachers who have many years ahead of them. One of the methods used to raise teachers’ awareness of professional achievements and increase this success is the teacher’s self-evaluation. This evaluation process will enable them to increase their motivation and creativity, be reflective thinkers, be aware of their shortcomings, and find ways to eliminate them and strengthen themselves.

It is seen that the educational alternatives of teachers who are trying to improve themselves are increasing with the developing technology. Most of the teachers who participated in the research stated that they examine good examples to improve themselves professionally. It has been observed that teachers prefer to use social media channels to reach good examples. Innovative teachers’ task perception allows students to work independently and have more responsibility for their learning. However, teachers who have difficulties accepting new innovation goals put more responsibility on themselves and strongly believe in their expertise as teachers [1]. As an evaluative component, task perception is the normative component of self-understanding. This component includes their ideas about their duties and responsibilities to be a good teacher [41]. There are many studies in the literature that have reached similar findings [30, 42, 44, 49, 52]. This situation shows how online education platforms such as Education Information Network (EBA) (Education Information Network [EIN]) offered by the Ministry of National Education in Turkey can play a key role in terms of the professional development of teachers and student’s education. It can be said that teacher-student communication will take on a more informal structure than its formal structure at school through social media platforms, which occupy a large place in students’ lives. This situation will lead to more comfortable communication by eliminating communication walls between students and teachers in school and educational processes.

In the light of all these findings we obtained in the research, the concept of professional self-understanding is closely related to the emotional context. As a result of the depersonalisation that occurs with emotional exhaustion, individuals now lose their ability to empathise with people and events. For this reason, teachers need to increase their interest in the profession and develop positive attitudes to have a high level of self-compassion. However, in our study, it was observed that teachers were reluctant at this point. Nearly half of the participating teachers stated that their current position/situation seems sufficient for them and expect to retire in this way. It is noteworthy that teachers at all stages, except for the career entry stage, do not put forward concrete expectations for the future (future perspective). Only teachers at the career entry and calmness stages plan an academic career. This situation can be interpreted as the excitement of teachers who started their profession with great hopes, professionally and individually/academically, decreased over time. The future perspective includes a time element for self-understanding. The future perspective reveals a teacher’s expectations about the future of their professional life [41]. For example, the future-oriented perspective of teachers with innovative goals seeks to take on a different role within the organisation and expects to play a role in designing important educational developments in collaboration with their colleagues. Teachers who have difficulties accepting innovations desire to limit their duties [1]. Teachers think that their expectations about the profession are not met at the desired level, they are worried about the future, and the teaching profession is not respected compared to the past [31]. Similarly, one out of five teachers who participated in our study stated that negative attitudes towards the profession have developed, especially in society. They stated that they are intimidated to do this profession and do not have hope for an improvement in this matter, even if they are still young.

### Recommendations

When the relationship between social status, acceptance, and individual motivation is considered, society’s negative judgments about the profession make individuals reluctant to choose this profession. However, it is seen that teachers who still practice the profession are also negatively affected by this situation in terms of creating professional career plans. Studies should be carried out to restore the eroded reputation of the profession in the eyes of society, which is voiced as a prophet’s profession. This profession should be provided with much more attractive opportunities in economic terms. Finally, the research was conducted with teachers working in a metropolitan city like Istanbul. Comparing the findings by conducting similar studies with teachers working in rural areas will be important in revealing whether teachers’ professional self-understanding differs in terms of the quality of the settlements where they work.

### Limitations

There are some limitations of the study. First of all, the study was carried out with the participation of teachers working in Pendik district of Istanbul province. It is thought that the results will be more inclusive if teachers working in different districts or provinces are included in the participation. Secondly, there was no equality in the gender variable from the demographic characteristics of the participants. The fact that women were determined as more participants than men in the study can be considered as a limitation. Third, because the collection of study data coincided with the Covid-19 pandemic process, some respondents were uncomfortable contacting them directly. For this reason, it can be considered as a limitation that the participants wanted the interview to be short.æ

## Supplementary Information

**Additional file 1.** Interview Transcripts with Teachers.

## Data Availability

The authors indeed provided all raw data on which the study is based. All data generated or analysed during this study are included in this published article [and its additional files].
