# Supplementary material for: Professional self-understanding of teachers in different career stages: a phenomenological analysis
Source: BMC Psychol. 2022 Mar 7;10:57. doi: 10.1186/s40359-022-00769-w (PMC8900645; doi:10.1186/s40359-022-00769-w)
Supplement: Supplementary file 1 — Additional file 1. Interview Transcripts with Teachers. [file 40359_2022_769_MOESM1_ESM.docx]

**PROFESSIONAL SELF UNDERSTANDING**

**QUESTION 1: How would you describe yourself as a teacher? (What kind of teacher am I? How do I describe myself as a teacher?**

P-1: I am a teacher of my own. I like to research. I also force my students to do research. I like to study, not intelligence.

P-2: Innovative, open to development, giving importance to student-centered teaching.

P-3: I am an idealist teacher, I want to have salt in the soup so that the country, family and children can be successful. I am not very knowledgeable, but I try my best. I am very happy that my environment is successful.

P-4: There has been a change in my understanding of discipline compared to the first years I started working. I always tried to be the best. I think this feature of mine has affected my professional life as well. I've worn out my child. But I don't regret it.

P-5: I am a self-sacrificing and self-sacrificing teacher. I do not come to class unprepared.

P-6: I am a teacher who tries to do his job well. I am a kind of teacher who gives importance to in-class discipline, who cares about classroom discipline, who cares about and considers student psychology. I am an open teacher.

P-7: I had never thought of such a thing as teacher types until now. But as a teacher, I see myself as a theater actor in the classroom. My role changes depending on the situation, event, person. Sometimes it is authoritative, tough, determined. Sometimes it is indecisive and timid. But the truth is that I try to see the school through the eyes of a student. I try to approach with empathy.

P-8: I am a teacher between sweet and hard: I am a teacher who sets the rules when appropriate, is compassionate and affectionate when appropriate.

P-9:To myself; I can describe myself as a disciplined (balanced) teacher who tries to be better than my past, accepts his mistakes, tries to be beneficial to my student. I am open to communication. I put my egos aside. I try to establish a good emotional bond with my parents and students. I think that feelings will lead to better learning.

P-10: I think I am lacking in some issues. I am constantly exchanging ideas and researching new things to make up for my deficiencies.

P 11- I am a teacher who loves innovation. I am a teacher who advocates learning fun by playing games.

P-12: I would describe him as someone who is disciplined, who cares about his students, knows his students' psychological, family, and financial situations and acts accordingly.

P-13: I define myself as a teacher who is researching, open to innovations, learning while teaching, enjoying what he is doing, open to communication, loving to produce, and aiming to bring his students to a level that can be self-sufficient in any environment.

P-14: I am a partially successful teacher who tries to improve himself and tries to adapt to changing conditions.

P-15: I am a teacher who tries to treat his students equally and always strives to complete the goals I have set.

P-16: I think that I am a teacher who is interested, caring and able to learn new things from students.

P-17: As a physical education and sports teacher, I define myself as a teacher who wants for my student what I want for my own children. I think this is very comprehensive.

P-18: He may not be a teacher, but an educator because of his branch. He is someone who tries to do his best, but sometimes sinks to the bottom and feels that he is beating water in the mortar, and then gets up and continues. But first, I am a teacher trying to realize myself.

P-19: I am a teacher who listens to his students, tries to understand them, wants them to be good individuals and strives for it.

P-20: I am a concerned, hardworking, student-centered teacher. Business-oriented, open to new education-teaching approach, productive.

P-21: I am a teacher who believes that it is important to enter the lesson prepared, knows the importance of the feeling of admiration from his own student life, puts this feeling forward in the student and acts to gain his admiration and respect first, thinks that listening to music and talking about art in the lesson is effective and using them frequently in his lectures. .

P-22: My teacher for 20 years, my excitement has decreased. I think I can act professionally in the classroom. I am not too friendly to the student, I am not too far away.

P-23: I am a teacher who wants to teach students to question, comprehend the events and develop their powers of interpretation. I want them to perceive and comprehend all the processes around them and form their own ideas. I try to create individuals who think and perceive by saving children from rote patterns.

P-24: Success-oriented, who loves his job, wants to see more respectful students and parents.

P-25: I think I am competent in my profession. I like to research and develop myself. I am tolerant and caring towards my students.

P-26: I like to use different lecture techniques in the classroom. I stick to the curriculum. I stick to the annual plans. I have an authoritarian structure that encourages students to express themselves. The most important thing for me is to keep the student's curiosity alive.

P-27: I think that I am a teacher with the necessary equipment in the field. I am consistent in terms of work discipline and classroom control. I have deficiencies in student communication and motivation.

P-28: I am a teacher trying to be a guide. I continue to learn from my students. I still cannot say "I am now". Even though I do not want to be in a mold, I see that I cannot go out of the mold.

P-29: I think that I am an innovative and creative teacher who is open to learning.

P-30: I define myself as a teacher who is open to improvement, constantly renewing himself, researcher, disciplined and regular.

P-31: I am a teacher who is open to communication, acts in a planned manner, has goals, pays attention to his communication with parents and students, and tries to gain behavior as well as transferring academic knowledge.

P-32: A teacher who is idealistic, but struggles not to despair as the value given to the teacher decreases day by day.

P-33: I think that I have taught my lesson well. However, I do not think that I follow the technology sufficiently. I think that I treat students equally.

P-34: I am a teacher who can establish a relationship of love and respect with the student, aims at education and loves his job.

P-35: I am a self-developed teacher who has mastered his profession.

P-36: Knowledgeable in their field, competent in risk management and classroom management, disciplined, knowing and applying the regulations, standing equidistant from students, looking at students objectively, doing their job properly and on time, attending class on time, wanting the student not to be victimized, sincere towards students I am a teacher.

P-37:24 Since I worked for the past year, I can say that teaching is not a profession that can be done unless you love it. I love teaching very much. This profession is my dream job. I love students and school. I think that makes me a good teacher.

P-38: I am a teacher who likes to lecture and interact with the student in the classroom. I would like to teach students to question. However, I cannot always allow this to do my job well. I only want to tell the subjects that I like and find necessary. I can be harsh sometimes. Rather than hardworking students, master and responsible students attract my attention.

**QUESTION 2: How successful do you find yourself as a teacher? How do you evaluate your success? (How successful am I as a teacher? How good am I at my job as a teacher?**

P-1: I believe that the concept of success is relative. Therefore, we are successful when we match what we can give our students or what we want to teach. Therefore, I am a moderate teacher.

P-2: I am not a successful teacher. However, I try to reach my students by trying to touch their lives, try different methods to make them love my lesson and guide them to assimilate the course content.

P-3: First of all, I don't think it would be objective for us to answer this question. I don't find myself successful at all. But I try to be successful.

P-4: I can't evaluate myself in terms of success, I don't know how much success is compared to someone else. However, when I see myself as incomplete, I sit down and work. I don't like to be lacking, I think where I went wrong when my child doesn't understand. If I was successful, everyone in the class would be at the level I wanted. I couldn't achieve this in 21 years.

P-5: Success is relative. I cannot say that I am successful. I could not raise ideal students that I targeted and desired.

P-6: I find myself successful. Because I love my profession. I try to avoid behaviors that will negatively affect the future school life of my students and try to make them like the school. I believe that I have succeeded in this.

P-7: Do I find them successful? It is discussed according to their criteria. I have not had a student who could not read and write until now. If this is a criterion. I have students who graduated from very good universities. Of course, if I embrace their success. I think that counts as success.

P-8: I see it as successful. It is a very nice feeling that they remember me even when they grow up. I care very much that teachers keep in touch with each other.

P-9: I believe I am successful. I don't know the degree.

P-10: There are some factors that determine the success of the teacher. The student structure in the classroom and the parents affect this. I think that I behave honestly, openly, fairly, respectfully and equitably towards students in the classroom.

P-11: I find myself successful compared to the year I have been in the profession. Of course, I have some shortcomings. I try to complete them by researching and renewing myself.

P-12: I find it successful enough. I find myself successful because I do enough of the requirements of my job.

P-13: Although there are times when I am not completely successful, the positive feedback from parents and students makes me say I am good at my profession.

P-14: We have a lack of students' learning, internalizing and using what they have learned.

P-15: I think that I am successful as much as possible.

P-16: I am successful, but not enough. It can be done better.

P-17: First, I focus on raising good people. I follow all the innovations and try to teach them the best.

P-18: I can't say that I am very successful. But I do not know what success means here, what are the criteria for being successful. I see my work as abstract works. But I believe that I have done my best.

P-19: For me, the first indicator of success is the ethical changes in the student. I consider myself successful if the student can be more consistent and responsible.

P-20: No matter how hard I try for the vocational high school students at my school, I am not motivated because I can't see the results. In this sense, I cannot be said to be very successful. As a teacher, we always have shortcomings. I am not perfect. I do my best.

P-21: I think that I am a good teacher, but I know that sometimes I can fall short in my attitude and approach to students due to my decreased tolerance.

P-22: Considering the conditions we are in, I find it successful. If the group I work with is better, I can prove it. The things that the state wants me to give are not what the students here want to receive.

P-23: There are limits to my profession. It is very difficult to instill new ideas other than social truths in students coming from different cultures and family structures. Even transforming the society's perspective on women is very challenging due to the ideas of the young people who grew up in this social structure.

P-24:Success is not a one-sided thing.

P-25: I see myself above the middle.

P-26: I think I did my best. I make up for the problems and mistakes that arise from the conditions of the curriculum, students, and school environment as much as I can. I complete the curriculum in order to maintain equality of opportunity. For these reasons, I find myself successful.

P-27: I am successful in knowledge transfer and exams. It develops students in areas such as TYT, AYT, etc. I think that I have fully achieved the success in academic development in art awareness and in guiding students in intellectual fields.

P-28: I see education and training as a whole. I feel that I can touch my students. In this sense, I think I am successful, but it is never enough.

P-29: As a teacher, I find myself successful. However, I also know the areas where I failed. I evaluate my success not with my other friends in the profession or student success, but with my self-development and what I add to myself every year.

P-30: Although I am in the second year of my profession, I consider myself more successful than normal in terms of the studies I have done and the schools I have worked at.

P-31: I find myself successful when I evaluate the feedback and satisfaction situation I receive from the stakeholders under the roof of the school. However, there are differences between the teacher role I played in the first years of my duty and my current teacher role. I am trying to continue my profession by learning from mistakes and shortcomings.

P-32: He is a teacher whose academic success is ignored because we cannot work in project schools, but he is a successful teacher according to his own conscience.

P-33: I am successful. But I am also lacking. As the interest of the students decreases, my success decreases. Because my enthusiasm to study decreases. I cannot renew myself.

P-34: My teaching life was not very unsuccessful. However, I cannot say that I am a successful teacher. I try to fulfill the given task perfectly. I follow the innovations in education closely.

P-35: I find it very successful, I am very good at my profession.

P-36: I think that I am a successful teacher in the eyes of the student. The student is the mirror of the teacher. If the student is happy in the lesson, I am successful in that lesson.

P-37: I would like to compare our achievements with student achievements, but since I am a vocational high school teacher, students do not seem very successful when they come to us. But I think that I am a good teacher and I am successful.

P-38: Success is a variable concept. It depends on what we achieve. For this, I need to know what you are asking. But I am 50% successful in students who do not have the enthusiasm to learn. In order not to lose those who are enthusiastic.

**QUESTION 3: Why did you choose the teaching profession? What are the factors that motivate you in your profession? (Why did I choose this profession? What motivates me in my profession?)**

P-1: I chose the teaching profession willingly and lovingly. I think that my motivation increases when my students come to a place as a career.

P-2: It is an invaluable phenomenon to touch people's lives and see the development of a student in their life and that you have a small part in it.

P-3: I knew myself, so I started primary school, I said "I will be a teacher". Fields have changed over the years, but my desire to be a teacher has never changed. (First I wanted to be a history teacher, a geography teacher, and mostly physical education). What motivates me is meeting a successful student friend after years.

P-4: After the university exam, all of my choices were teaching. My brother, whom I taught during my student years, would have children next door. Maybe I grew up as a teacher. I didn't think of a different profession.

P-5: There are two factors in my choice of teaching profession. One is that I am not the child of a farmer family. I do not want my hand to hold bread as soon as possible. The other is that I admire my primary school teacher. When I finished primary school, my father did not want to send me. My teacher insisted on my father many times.

P-6: I decided to become a teacher in primary school. I loved my class teacher very much, I used to imitate him. Also, I love children, touching their lives and bringing in newly educated individuals to society makes me happy.

P-7: It is not a planned choice. Let's call it destiny. Positive feedback from my former students and positive words from my parents motivate me.

P-8: The positive energy coming from the children makes them happy. We can see their growth with their mothers and fathers.

P-9: I chose this profession because I love children. I like to explain things to people. I like to talk. Friendly and grateful people motivate me. The people in my organization motivate me.

P-10: I love this profession. I love children.

P-11: I love children and I chose this profession thinking that I am patient enough. Their conversations and love motivate me.

P-12: I chose it because I love children. Teaching students knowledge and good behavior motivates me.

P-13: Actually, I did not choose this profession in the beginning. Let's say it coincided with the ranking in the university exams. However, after I started teaching, I realized that this job was completely suitable for me. As for motivation, I think the goals related to my class, my friendships at school, a constantly moving life and the sincere and sincere behavior of young students are triggered.

P-14: Sharing my knowledge, benefiting from my experience, satisfying my need to be useful.

P-15: I wanted to be a teacher in general. I don't think there is much I can do. However, since I was assigned from a different field, I had to start.

P-16: I have always liked the idea of ​​teaching students new information. Experiencing this excitement with the student is my biggest motivation. In addition, getting the opportunity to touch people's lives is one of the important factors for me to do this profession.

P-17: I chose it on my mother's word. "If you do not do this profession, you will not be able to find a teacher for your children. Your job is very important for the country," he said.

P-18: It's all about the preference system. Motivating things in my profession, student statements, feedback from students.

P-19: I think it was because of my teachers who influenced me as a child. Being able to transfer useful information that I know to others motivates me.

P-20: I became a teacher because I love to tell, teach and share information. I think teaching is about learning new things every day and realizing how little I know. I like to learn and share what I have learned.

P-21: There are many teachers in our family and relatives. Especially my father was a very respected and good literature teacher in his profession. My reason is my father.

P-22: In our period, there were not many options with job guarantee.

P-23: As Atatürk said, raising generations with a free conscience was my biggest dream. Even seeing the change in behavior and opinion of a few of my students each year motivates me. Because even a few people can change society.

P-24: As long as the communication with the students is good, I will be motivated.

P-25: Because it is different from other professions. Because I love teaching.

P-26:1) I like the field of history.2) Transferring information, reading, discussing, thinking analytically, and establishing long-term relationships with people are suitable for my character3) having a job guarantee 4) I graduated from a teacher's high school. I am motivated by students' curiosity, their interest in the past and today's problems, and their success in university exams.

P-27: I come from an education-related family. I was very interested in literature during my high school years and I chose university in this direction. I do not like genres such as poetry, novels and stories, artistic language, and imagery make me happy. Giving my students the pleasure of language and literature; It is a pleasure to introduce poetry and teach the language.

P-28: Honestly, I did not start out as a teacher. I didn't like it at first. However, over time, seeing the light in my students' eyes became my biggest motivation.

P-29: Teaching was the last of my university choices. It was my only teaching choice. So it was a forced choice. Even though I chose the profession because I had to, I try to improve myself because I try to do what I do successfully. Learning new things and encountering new processes motivate me in the profession.

P-30: I graduated from Science High School. Normally, I didn't want to be a teacher. In fact, I wanted to take the university exam again and change and I won another university. Being able to touch the students and add something new and their feedback are the factors that motivate me the most.

P-31: I chose the teaching profession with the guidance of my family. The things that motivate me in my profession are that I love children very much, seeing progress and change in students, being appreciated by the stakeholders for what I do.

P-32: It was my dream job. Everything that motivates me is decreasing day by day.

P-33: Due to the conditions of the period, I chose this profession not voluntarily, but out of necessity. I currently do not have a student profile that motivates me. Very rarely do I survive with some aspiring students and graduate students.

P-34: It was my childhood dream. There are 4 teachers in my family. I studied at a teacher's high school. My only motivation in my job is the students who come to visit me.

P-35: The reason for choosing to be a teacher is because I don't like teaching. Motivating elements; quality students, a good educational environment, managerial appreciation, student feedback.

P-36: I like lecturing and teaching. A good student always motivates the teacher. A good syllabus always motivates the teacher. time spent at school motivates the teacher. Friendships at school motivate the teacher. Being in harmony with the school administration motivates the teacher.

P-37: It was my ideal job. I think that my uncle's being a teacher may have caused me to choose this profession.

P-38: I love and respect. A peaceful work environment and happy students motivate me.

**QUESTION 4: What/what are you doing to be a successful/good teacher? (What do I do to be a good/successful teacher?)**

P-1: I like research. I love doing this both in my course and in terms of legislation. That's why I think I'm successful.

P-2: I try to participate in the trainings and symposiums I can reach in order to renew myself.

P-3: First of all, I love my job, I always come to school with excitement. I update the topics as much as I can. I communicate with my children on patience, love and interest. I am very happy to listen to them. I learn a lot from them.

P-4: I evaluate myself. I'm trying to make up for my shortcomings.

P-5: I read books about teacher-student relations, dialogues and behaviors. I am researching lecture techniques. I am in search of a better narrator. I try to teach the lessons interactively. I support it with animation course materials. I reinforce it with examples.

P-6: In order to be a good teacher, I read educational works as much as I can. I follow good examples on social media. I benefit from the ideas and practices of my experienced colleagues.

P-7: I observe my fellow teachers around me. I follow educational sites and fellow teachers on social media. I watch all the movies I can find with education. I get information about education-school-teachers-friends (school) with all the children I can communicate with.

P-8: I try to follow the technological developments. I attend seminars. I try to work collaboratively with other teachers.

P-9: I try to be better than yesterday. I follow the agenda. I try to learn things that I don't know. I try to improve myself in communication. I read books, follow technology, and use it. (Usefully)

P-10: I strive to raise students who respect people, love their homeland, and know what they want.

P-11: I am always researching. I am trying to develop materials. I embody learning up to 75%. I guess that works too.

P-12: I am constantly educating myself. I try to get to know the students in every aspect.

P-13: I am doing my job. I pay attention to doing it right and working in a planned manner. I'm doing research. I read books about education. I'm trying to find out where I'm missing. I am determined, I never give up. I attach importance to parent-student-teacher communication.

P-14: I face my shortcomings, I accept change, I try to teach more, not to know more.

P-15: I try to keep my knowledge fresh. I follow the latest on professional matters.

P-16: I do not stop learning.

P-17: I attend certificate programs from universities in the summer. I am reading books.

P-18: I am reading a book. I'm trying to lower my expectations. I'm trying to see how valuable it is to teach or gain a little something.

P-19: I follow current issues related to my branch. I extract important points from each of the trainings held during the seminar periods and try to implement them. I try to stay positive.

P-20: A successful and good teacher is a relative concept. A teacher is open to learning, and a researcher is successful. Of course, this changes depending on the school and motivation resources. I do research on my field and read scientific articles. At the same time, I try to learn new teaching approaches and apply them in my classroom.

P-21: I read, I listen, I explain, I try to communicate.

P-22: I use some technology, I try to be up to date.

P-23: To improve myself, I read books and watch documentaries about my field. I try to make the subjects more enjoyable, I try to make them perceive by using lots of visual materials. I'm trying to develop their powers of interpretation.

P-24: I follow current teaching methods and approaches.

P-25: I listen to my students. I am doing research on my field. I communicate with other colleagues. I use their ideas.

P-26: I am a good reader in my field. I care about keeping myself alive. I choose the lecture methods and techniques that I will use in the classroom by talking with the student. I aim for students to be ready for the lesson with a short research assignment.

P-27: I follow the development and change in my field. I follow the questions, language and changes in ÖSYM and MEB exams. I read and know current literature.

P-28: I try to follow the agenda. I try to get to know and understand my students. If I understand them, they can love me. If they like me, they will also be successful academically. It is important to come to class prepared.

P-29: I try to learn new things so as not to repeat myself. I am trying to improve the techniques and methods I use while teaching. I try to outdo myself, I try to produce more than the previous year every year.

P-30: In the future, I aim to be a teacher in the field of education, where I can come from the field and make important decisions and introduce significant innovations into the system, both as an academician and as a teacher in the ministry.

P-31: I continue my profession as a school administrator. I didn't have big goals like making a career or getting promoted. I strive to provide the best service for the institution I am managing. I encourage and support my employees in this direction. I think that this sacred profession should be done with qualified teaching staff and that comprehensive education reform is needed for this, and that the greatest wealth for a country is the awareness that qualified citizens are connected to its culture.

P-32: I don't expect much as long as this worthlessness continues.

P-33: I plan to spend the last years of my job as an administrator.

P-34: My future career goal is to learn teaching practices by living abroad for a while and apply them when I return to my country.

P-35: Academician.

P-36: It is my expectation from the future to teach with better students.

P-37: I am where I want to be.

P-38: To continue as a teacher.
